# Evolution in Lithography Techniques: Microlithography to Nanolithography

**DOI:** 10.3390/nano12162754

**Published:** 2022-08-11

**Authors:** Ekta Sharma, Reena Rathi, Jaya Misharwal, Bhavya Sinhmar, Suman Kumari, Jasvir Dalal, Anand Kumar

**Affiliations:** 1 Deaprtment of Physics, Chaudhary Ranbir Singh University, Jind 126102, India; 2 Department of Physics, Maharani Kishori Jat Kanya Mahavidyalaya, Rohtak 124001, India

**Keywords:** lithography, resist, mask, nano-devices, alignment marks

## Abstract

In this era, electronic devices such as mobile phones, computers, laptops, sensors, and many more have become a necessity in healthcare, for a pleasant lifestyle, and for carrying out tasks quickly and easily. Different types of temperature sensors, biosensors, photosensors, etc., have been developed to meet the necessities of people. All these devices have chips inside them fabricated using diodes, transistors, logic gates, and ICs. The patterning of the substrate which is used for the further development of these devices is done with the help of a technique known as lithography. In the present work, we have carried out a review on different types of lithographic techniques such as optical lithography, extreme ultraviolet lithography, electron beam lithography, X-ray lithography, and ion beam lithography. The evolution of these techniques with time and their application in device fabrication are discussed. The different exposure tools developed in the past decade to enhance the resolution of these devices are also discussed. Chemically amplified and non-chemically amplified resists with their bonding and thickness are discussed. Mask and maskless lithography techniques are discussed along with their merits and demerits. Device fabrication at micro and nano scale has been discussed. Advancements that can be made to improve the performance of these techniques are also suggested.

## 1. Introduction

The comfort, healthcare, and transportation of modern society are significantly dependent on the development of electronic devices. The expectation of consumers to get smaller, high speed, and much less energy consumed per computing function electronic products at lower prices are enabled by reduction in chip sizes and high throughputs. In this course, patterning of semiconductor material is one of the basic requirements, which is done by the technique known as lithography [1]. Lithography is not only the driving technology in semiconductor and IC industry, but it has an important role in the fabrication of commercially available microelectromechanical system (MEMS) devices [2]. It is also used for prototype fabrication in developing nanoscience and engineering applications [3,4,5]. Some commercial MEMS devices are MEMS accelerometers installed in electronic devices and automobiles, airbags sensors, optical switches, microphones, projection display chips, and biosensors. MEMS pressure sensors are used for detecting pressures in car tires [6] and blood vessels. To name a few, nanoelectronics for denser and faster computing, nanomedicine for diagnosis and treatment of many diseases including cancers, cardiac disease [7,8] and Alzheimer’s disease, nanoelectromechanical systems for high-sensitivity and high-resolution sensing and manipulating and nano-biosensors for ultra-low-resolution sensing and manipulating have all contributed to traditional technologies by unlocking alternate routes to counter current technical barriers. Electronic devices such as CMOS, NMOS, GaAs PHEMT, MOSFET, DRAM, MPU, and flash memories are successfully fabricated with the help of lithography at a nanoscale [9,10].

On a semiconductor substrate, a variety of processes is performed to fabricate a chip which is then wired together to form an integrated circuit (IC). It is estimated that about 30% of the cost of manufacturing a chip is accounted by lithography, which makes it a prime part of manufacturing of ICs. As the chip density is increasing with time, this technique is a technical limiter in reduction of the size of the devices. The shapes, dimensions, and location of various components of an IC are established by the design process in the development of a device. The conventional photolithographic process uses UV light exposure (350–430 nm). Diffraction effects limit the minimum device dimension, so new lithographic techniques will be required to achieve the dimensions and the accuracies required for future generations of devices. Recently, various advanced lithographic techniques have been introduced including short-wavelength photolithography, electron beam lithography [11], X-ray lithography, and ion beam lithography [12]. Final choice for the selection of best lithographic technique will depend not only on the performance capabilities, resolution, and registration accuracy but also on the cost involved in manufacturing of integrated circuits.

Some of the common constitutes of lithography are optical system, resist, mask, and alignment marks. Optical system consists of lens, source of illumination, light detectors, image plane, etc. Lithography simulators can be used to investigate and optimize various illumination and lens conditions. Resists are the materials that change their properties when exposed to light in response to the incident photons. Resists are composed of film-forming resin, a solvent system (for the deposition of film), a photo initiator (for modification of the dissolution rate of film in a developer solution after exposure to light), and additives (for performance improvement). In general, there are two types of photoresists: positive and negative photoresists. In positive resists, the exposed region becomes more soluble due to which it gets removed in the developing process. The patterns formed are the same as those on the mask. Whereas in negative resists the exposed regions become less soluble due to which the patterns formed here are the reverse of the mask patterns. Both are used in the semiconductor manufacturing industry but positive photoresists are mostly opted by the semiconductor suppliers due to their higher resolution capabilities. But negative resist is not necessarily a thing of the past. Most commonly used resists are acrylate-based resists such as PMMA [13,14].

Further, the first pattern transferred on a wafer contains a set of alignment marks [15]. Mainly four types of alignment marks have been recognized: trench marks, metal marks, damascene marks, and combo marks. A few marks that are used in ASML scanners are AH11, AH53, AH32, and AH74. The grating period is 16 µm and 17.6 µm for these marks [16]. ASML has also developed the front to back alignment (FTBA) functionality to meet the demand for high throughput lithography. This system is named 3DAlign^TM^. With the help of two optical branches, the back side marks are projected to the front side. FTBA lithography is extremely important for the manufacturing of micro electro mechanical systems (MEMS) or micro opto- electro mechanical systems (MOEMS) [17]. A new set of alignment marks known as ATHENA has been designed by a joint development program between ASML and Motorola, to fulfill the requirement of least mark size. A new mark design reflective integrated plateau (RIP) has also been designed by ASML. These new marks are designed so that the stability of signal strength of alignment marks could be increased and an improvement in the overlay capability and stability could be seen [18]. By reducing mark damage noise, better alignment accuracy can be achieved with the help of some alignment marks such as ASML AH53 and AH74 which have high odd-order diffraction light. A new alignment mark ASML SMASH has been designed to customize the alignment mark [19].

Masks are made up of glass or quartz plates having a coating of hard surface materials such as chromium or iron oxide. The pattern is generated on the mask with the help of a pattern generator. The generated pattern is transferred to the resist on the wafer. Phase shift lithography can be used to get very high resolution [20]. All phase-shifting masks have a phase shift layer with a phase shift of close to 180°. The phase-shifting mask shifts the light with a half wavelength. Depending upon the mask, lithography techniques are divided into two parts: masked lithography and maskless lithography. In masked lithography, a large area of wafer is exposed simultaneously and patterns are drawn over a large area, which results in fabrication of high throughput devices, up to tens of wafers/h. High throughput allows the fabrication of high-density devices. Lithography includes optical lithography [21,22], extreme ultraviolet lithography [23,24], and X-ray lithography. However, due to the presence of mask, there are more chances of defects due to mask damage which limits the resolution of the device. The resolution is improved by reducing the wavelength of illumination and by increasing the numerical aperture of the lens used. Maskless lithography includes electron beam lithography [11] and focused ion beam lithography [12]. In this technique, patterns are drawn onto the wafer in a serial manner which allows high resolution up to nanometers. As it is a serial process, different shape patterns can be drawn by this technique. However, it exposes only a small portion of the wafer in one step reducing the throughput. Due to its low throughput, it is not suitable for mass production.

**Figure 1:**
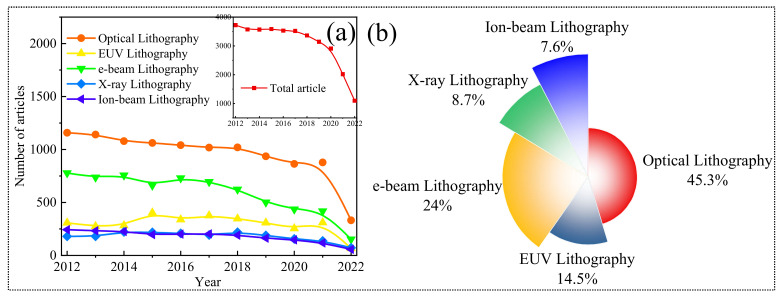
(**a**) Trends in lithography and its various techniques and (**b**) comparison of work done on different lithography techniques in the last five years [Data source: Scopus].

A lot of work has been done on lithography in the past decades. Figure 1a shows the trend in lithography and various lithographic techniques in the last ten years. Figure 1b depicts the comparison of the work done on various lithography techniques in the last five years. These data show that the demand for lithography is continuing in the semiconductor industry. Instead of the development of advanced lithography techniques, optical lithography is still trending at the top. The work on lithography is decreased in the last few years because new digital techniques which give sharp and precise patterning in a short time are developing for the fabrication of nanoscale devices. The demand for optical lithography and other conventional techniques is decreased with the evolution of new lithography techniques such as nanoimprint lithography, coherent lithography, atomic layer deposition lithography, photon upconversion lithography, and chemical-based direct self-assembly (DSA) which are rapid patterning techniques scaling down the feature size up to 10 nm. Among the different lithography techniques, optical lithography, extreme ultraviolet lithography, electron beam lithography, X-ray lithography, and ion beam lithography which are conventional lithography techniques, are discussed in the present work because these techniques consist of similarity in the set of constituents except in the exposure system. These techniques are also similar in the process flow of patterning.

## 2. Optical Lithography

In the manufacturing of microelectronics by the semiconductor industry, optical lithography has become the most powerful and highest throughput pattern depiction process. It is widely used for integrated circuits, semiconductor devices, thick and thin film passive components interconnection and packaging. Photonic wires, silicon single-electron transistors (SETs), crystal circuits, and electron pumps compatible with CMOS can be fabricated on silicon-on-insulator (SOI) with the help of optical lithography. The n-type device fabricated using optical lithography gate-all-around (GAA) SiNWS MOSFETs with a width of 4 nm channel is functional at room temperature [25]. Graphene nanopatterning can be achieved using quantum optical lithography. An array of 20 nm multiple lines was written using optical lithography [26]. Recently, a single-emitter plasmonic patch antenna using spatial modulated light was fabricated using optical lithography [27]. The everlasting use of optical lithography is the direct result of its highly parallel nature, which allows a vast amount of information to be transferred in very short time. In optical lithography, large amounts of chips are manufactured at one time, so it is a time-saving and fast production technique and the cost of manufacturing is less. For IC fabrication, optical lithography using UV light (0.2–0.4 µm) or deep UV light is the most widely used technique. In optical lithography, ultra-small patterns are printed on wafers to make complex circuits, which drive today’s information explosion. The credit for extraordinary growth of the semiconductor industry is deserved by technological advances in optical lithography and it is the key enabler for scaling feature sizes of ICs.

**Figure 2:**
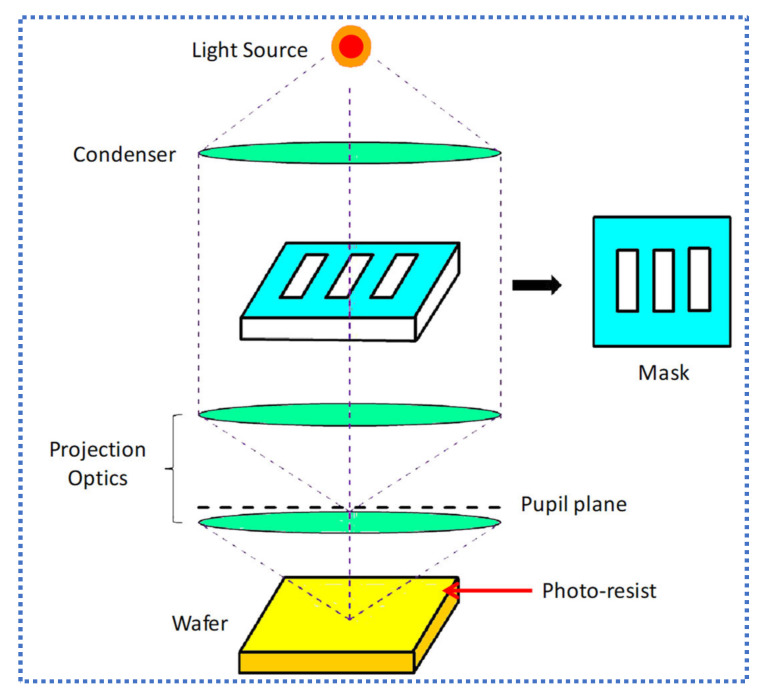
Schematic of optical lithography.

The early process used for optical lithography was contact printing. In this process, one-to-one mask contact was made. The major issue in this process was the high level of defect generation due to the contact between the mask and the wafer. To resolve this issue, 1:1 projection exposure was introduced. A schematic of optical lithography is shown in Figure 2. The resolution of the exposure system can be calculated using the Rayleigh equation,

(1) $$R=\frac{k1\cdot \lambda }{NA}$$

(2) $$DOF=\frac{k2\cdot \lambda }{NA^{2}}$$

where *λ* is the wavelength of the illuminated radiation, *R* is resolution, *DOF* is the depth of focus, *k*1 and *k*2 are Rayleigh constants, and *NA* is the numerical aperture.

A high numerical aperture (*NA*) or shorter wavelength is necessary to get high resolution. To achieve a high *NA* in 1:1 projection exposure system was very difficult. Attempts were made to reduce the wavelength but no compatible light source was available at that time in the deep ultra- violet region (DUV). Excimer laser was proposed to use in the exposure system, but no compatible resist material was available at that time [28]. At the same time, reduction projection exposure was developed at a rate faster than DUV because of the lack of development of resist materials for DUV. Higher *NA* can be obtained with the help of reduction projection exposure with a step and repeat system. The first commercial reduction exposure system was DSW (direct step on wafer). In the early stage of stepper, a g-line source of high-pressure mercury lamp was taken with a wavelength of 436 nm. A resolution of 1 µm was obtained with a *NA* of 0.28 in 1970s. The *NA* was improved to 0.5 in the mid of 1980s. But higher *NA* results in the loss of depth of focus at a fast rate. At the end of 1980s, i-line (365 nm) exposure was introduced after the g-line (436 nm). Attempts to introduce KrF excimer lasers (193 nm) were also made.

Dr. G. M. Dubroecq of Thomson CSF introduced an excimer laser-based exposure system in 1982 and a full field stepper was introduced by Dr. V. Pol in 1986 [28]. The projection optics of g- line and i- line were quite similar which helped in the development of i- line reduction exposure system. The projection optics of KrF laser was quite different from g- line. The lens of quartz material was required for KrF excimer laser. But the demand for high resolution was continued by the semiconductor industry. Attempts were made to reduce the exposure wavelength from 193 nm to 157 nm with the help of F_2_ laser. But it demanded a new set of exposure tools, resist materials, and mask materials, due to which this attempt failed. Then immersion lithography was introduced to achieve high resolution by reducing the wavelength. Dr. Burn J. Lin was the first to introduce water immersion lithography in 2003 by using ArF exposure system. A commercial immersion system was developed by Dr. S. Owa of Nikon named local-fill [29]. In this system, water was filled between the lens and the wafer. Later, a similar system was developed by ASML. With this immersion exposure system, the wavelength of 134 nm can be used to print the pattern at a *NA* of 1.35.

### 2.1. Exposure System

In the beginning, step and repeat reduction projection system was introduced. By reducing the magnification, the entire mask pattern was projected onto the wafer. After this, wafer was moved one step ahead and exposed the mask pattern again. Repeating this process, the entire wafer was exposed in steps. Therefore, this system was called stepper. Earlier, the reduction ratio was 10:1 which becomes 5:1 with an increase in chip size. This ratio was reduced to 4:1 by the scanner system. By using the scanner system, larger scanning field can be obtained with a smaller lens system. Further to increase the resolution, modified illumination technology was introduced at the light source. Conventional masks were replaced by phase-shifting masks and OPC (optical proximity effect correction) was applied. An immersion system was used between lens and wafer [30].

### 2.2. Resists

Resists are photosensitive materials, which polymerize or depolymerize during their exposure to light. The first positive-acting photoresist was novolac and diazoquinones (DNQ) photoactive dissolution inhibitors which were developed by Kalle and Company, AG in Germany and its American subsidiary Azoplate Corporation, in the early 1920s. The choice of resist depends on some factors such as resolution, cost, processing ease, etc. Due to high resolution and wide processing ease of negative resists, these are used to pattern the CMOS logic devices which are IBM’s advanced devices. Negative resists were introduced in the 1960s, in which the first negative resist was designed, which was based on free-radical initiated photo-crosslinking. KTFR, a system was introduced by Kodak, consists of bisaryldiazide with a cyclized polymer and polyisoprene which has adhesion properties in presence of UV light. Due to several disadvantages of this resist, in 1968, Dupont introduced some new dry-film photoresists [31], which are most widely used dry-film resists consisting of a layer of photopolymerizable which is sandwiched between a support film of polyester and a separator sheet. Throughout the 1970s, the development of these resists was done in chlorinated solvents, but now aqueous-based developable systems are available [14]. In the early 1970s, negative resists such as KTFR as described above were unable to meet the resolution requirements for semiconductor industries. Therefore, based on diazo chemistry, new positive material was developed having many advantages.

**Figure 3:**
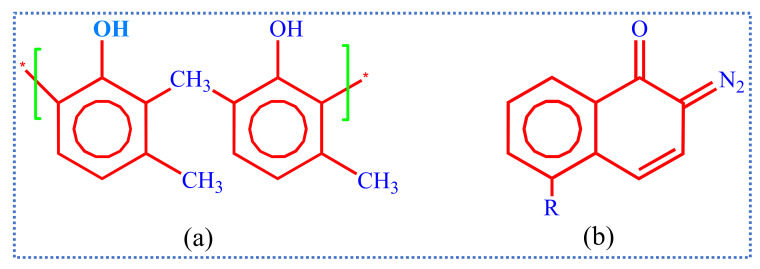
Chemical structure of (**a**) novolac polymer; and (**b**) diazoquinones photoactive compound used in most commercial i-line resist.

The most widely and commercially used resists in g-line (436 nm exposure wavelength) and i-line (365 nm) are novolac (a polymer having basic hydrocarbon ring with 2 methyl groups and 1 OH group attached), sensitized by DNQ (diazoquinones) as shown in Figure 3. Here the photoactive compounds (PACs) are DNQ which inhibits the dissolution of resists in the developer and get chemically changed when resist is exposed to light. After the chemical reaction, the developer does not affect the unexposed regions of resist, and a high-resolution image of the mask is produced if the exposed pattern accurately reproduces the mask pattern.

**Figure 4:**
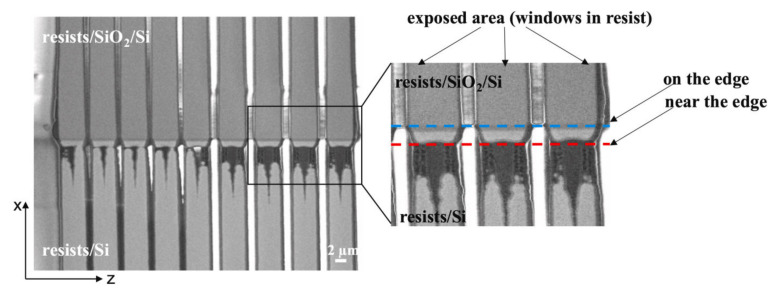
SEM image showing edge distortion of SiO_2_ step. Reprinted with permission from [35]. Copyright 2022, Elsevier.

In the early 1980s, with the development of optical tooling, there was a need to develop the resists because these diazo resists were not sensitive enough for future generation chips. In the late 1970s, some chemically amplified resists (CARs) were developed to meet future lithographic needs. Certain onium salts produced strong acids by photolysis, which could be used in designing new photo imaging systems. In 1982, the first CAR for DUV applications was reported by Ito and Wilson [32] which was based on an acid-catalyzed deprotection mechanism. Poly(4-t-butoxycarbonyloxystyrene), tBOC, and triphenyl sulfonium hexafluoroantimonate onium salts were the polymers used as first DUV negative resist system. In the late 1980s, when the tBOC was exposed to DUV light, it is used to manufacture 1 Mb DRAM [13]. Special carbon filters were installed to protect the CARs from the diffusion of contaminants. Both chemically amplified negative resists i.e., acid-catalyzed EPR and tBOC were capable for 1Mb chip manufacturing with patterning of 1 µm ground rules. Over the next 5 years, further developments in resists take place which were capable of manufacturing CMOS with 0.35 µm ground rules. Earlier used negative resists were developed in aqueous basic solutions which faced many problems; so, in 1986 Feeley [33] at Rohm and Haas reported a different approach in which acid-catalyzed condensation reaction takes place between an aminoplast cross-linker and melamine formaldehyde. Although a positive resist APEX gives 8% larger isolated lines than a negative CGR resist, currently for the fabrication of all 0.35 µm CMOS devices, this negative resist is being used and is commercially available from the IBM/Shipley DUV Resist Alliance. Epoxy materials have many applications in semiconductor industries; one of its derivatives bis-phenol, a novolac (SU-8), provides high-resolution patterning. PMMA is also a negative resist widely used in EBL, XRL, etc. Currently, acid-catalyzed cross-linking-based negative resists are used for the fabrication of critical 0.35 µm level of advanced CMOS chips [34]. The overall performance of photoresist should be estimated by resolution, LER (line edge roughness), and sensitivity. However, on non-polar surface, fabrication of devices in micro and nanoelectronics is still a challenge [35], as shown in Figure 4. On the edge and near the edge, the resist gets distorted due to exposure and other factors.

### 2.3. Limitations

With time, as feature size shrinks, optical lithography faces many physical and economic challenges. As the wavelength is getting smaller, lenses that are designed to focus the light absorb the light and a very less amount of light can reach the wafer. Optical lithography has some limitations such as it is diffraction-limited and is not applicable for curved surfaces. Photosensitive polymers are necessary for this technique, and masks needed for this technique are very expensive. Sometimes there is a mismatch between the lamp and resist [36]. To produce acceptable patterns, cost issues, overlay errors, and linewidth differences between exposure tools must be tightly controlled. As feature size decreases with time, a steady stream of improvements is required.

For 193 nm wavelength, optical lithography would go as far as 0.13 µm with *NA* = 0.65 and k1=0.35. For further resolution, immersion fluids were introduced within the gap between projection optic and wafer which increases *NA*. Currently, using water immersion ArF lithography, the IC industry is on the edge of manufacturing 45 nm nodes at 65 nm half-pitch. There are limitations in immersion lithography, i.e., fluids generate bubbles, leave strains, and carry particles around, which generate defects on the wafer which makes wavelength reduction not a viable option for the refractive system and hence researchers turn to reflective optics. If all the extension techniques i.e., high index material, solid immersion on the mask, and polarized illumination are successful and support k1=0.3 at 1.55 *NA*, governing to 37 nm half-pitch. The tendency of split pitch is used to reduce *k*1 to 0.15 from 0.3 and suggests a half-pitch of 19 nm. But the processing steps, tools, and masks in the split pitch technique are double, resulting in a loss of economy. That is why other lithographic techniques such as EUVL, multiple electrons beam direct write, etc., are introduced.

## 3. Extreme UV Lithography

**Figure 5:**
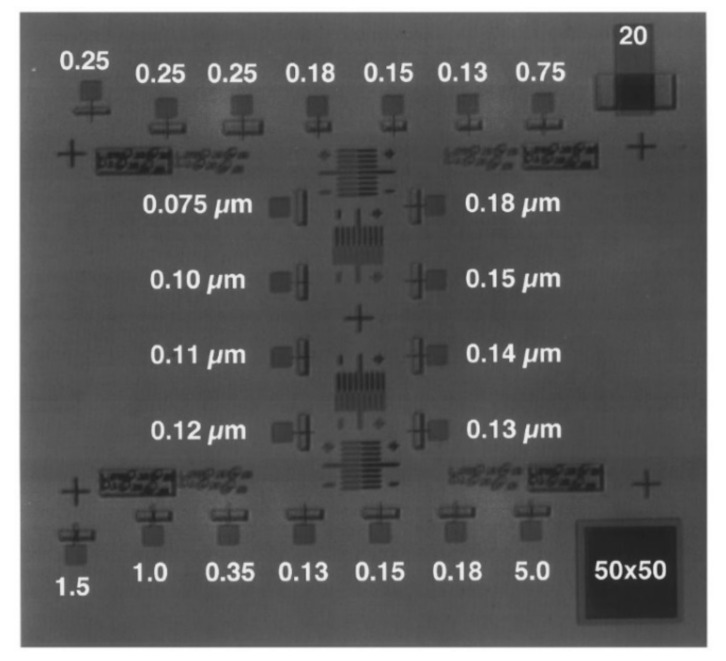
Configuration of NMOS device fabricated with EUVL gate-level. Reprinted with permission from [38]. Copyright 1996, American Vacuum Society.

Optical lithography can be used up to 100 nm integrated circuit (IC). For semiconductor devices < 100 nm, next-generation lithography is required. Extreme ultraviolet lithography (EUVL) which uses the 10–14 nm extreme ultraviolet light proposed in l988 can be used for IC fabrication at 100 nm. EUV technology is expected to expand below 30 nm. EUVL has been implemented in high-volume manufacturing (HVM) of the semiconductor industry since 2018. With the help of multi-patterning technologies, IC fabrication can be pushed beyond 3 nm [37]. EUVL is used for scaling of devices such as DRAM, MPU, and flash memory (NAND). NAND flash memory can be scaled down to 16 nm using this technique. NMOS transistors are fabricated by Sandia EUVL laboratory tool with a gate length of 1 µm as shown in Figure 5 [38]. Transistors of gate length up to 20 µm and capacitors of 50 × 50 µm^2^ were used in this process. EUVL can achieve high resolution with a single exposure and with fewer design complications and hence reducing the manufacturing costs. The use of low *NA* provides good depth of focus and avoids the use of proximity or phase shift corrector. The 4× masks used in EUVL are easier to write than 1× masks. As the size of the chip shrinks, EUVL provides reliability and economic yield, it also improves the quality of linewidths.

The main components of the EUVL system are an exposure tool, condenser multilayer mirrors, projection multilayer mirrors, and a mask. Two source concepts are mainly implemented: the electrical- (DPP) discharge-produced plasma source and (LPP) laser-produced plasma source. Tin and Xenon are used as emitting materials in both DPP and LPP. The conversion efficiency of DPP is high and has a low collecting efficiency because of the small solid angle. High conversion efficiency can be obtained with short laser pulses and high repetition rates. The conversion efficiency of the EUV source is determined by the efficiency of plasma generation and heating. Most efficient EUV sources imply various plasma densities, various plasma geometries, and various time scales. EXTATIC (EXTreme ultraviolet Alpha Tool Integration Consortium) studies the basic optical system of EUVL application. The partners in EXTATIC are various companies such as ASML, SAGEM, Carl Zeiss, and Xenocs [39].

**Figure 6:**
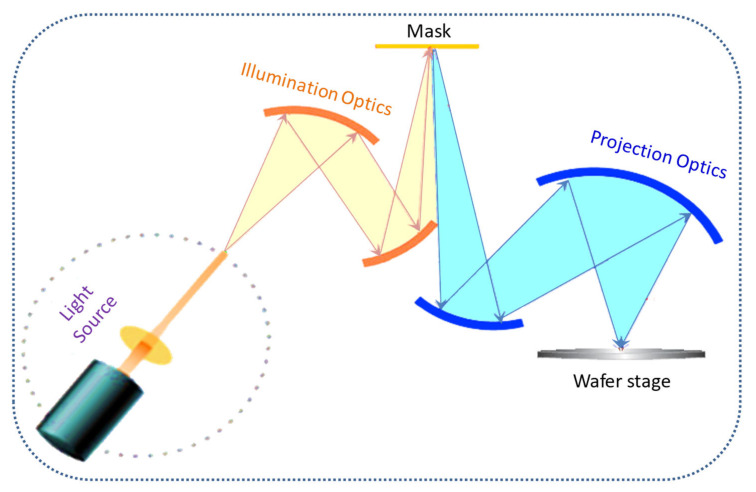
Set up for EUVL technique.

Reflective mirrors are used for the imaging capabilities of the scanner instead of refractive lenses used in optical lithography as shown in Figure 6. These mirrors are made up of silicon or molybdenum containing up to 100 alternative layers. For the 13.5 nm wavelength of EUV, the thickness of the Mo/Si layer is 0.75 nm. These mirrors reflect the light from the layers of the material by interference. With surface coatings, these mirrors can reflect a maximum of 72% of the EUV light falling on them. The rest of light gets absorbed by the mirror. The masks used in EUVL must be reflective and should not have a protective pellicle, as pellicle can result in absorption of the EUV beam [40]. A resonance-reflecting mirror of a narrow band is used for multilayer coating. There are repeated bilayer pairs in the multilayer material following the relationship; nλ=2dsinθ, where d is the bilayer space, *θ* is the incidence angle, and λ is the wavelength of illumination. For 13.5 nm EUV wavelength, 40 pairs of multilayer mirrors are used with a thickness of 271.5 nm, angle of incidence of 6°, and bilayer spacing of 6.79 nm. A commonly used multilayer mirror is made up of 40 Mo/Si layer pairs.

**Figure 7:**
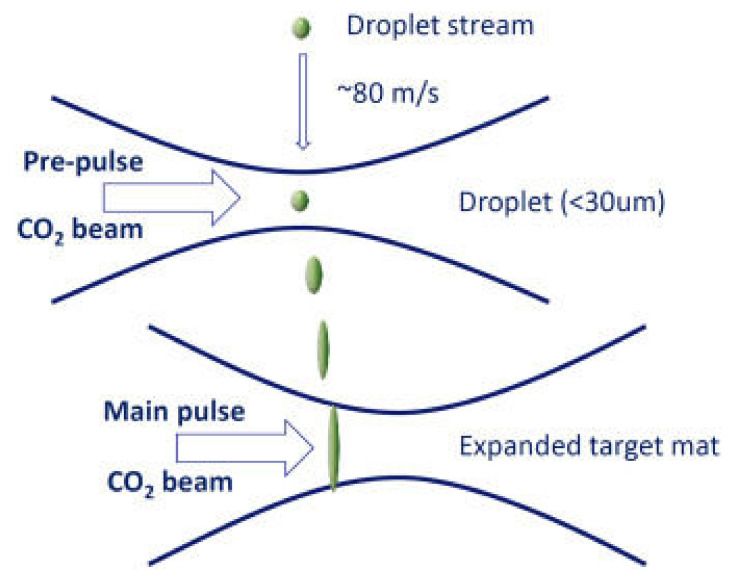
View of EUV generation and target formation. Reprinted with permission from [41].

Experimental investigation of EUVL started in the later 1980s. A spatial sight of generation of EUV and target formation is depicted in Figure 7 [41]. In the experimental studies of EUVL, to obtain a resolution of 200 nm, a synchrotron radiation source and a resist made up of polymethyl ethyl methacrylate (PMMA) were used. The laser-plasma source became successful in proving the practicability of the EUVL in 1991. In 1993, soft X-ray projection lithography and EUVL were declared same by the Optical Society of America.

In 1997, EUVL was expected to implement on a 65 nm node, but further advances in optical lithography pushed the implementation of EUVL beyond the 45 nm node. Full-field exposure tool was prepared by the EUV LLC in 2001 [42]. It consisted of a step-and-scan system, four mirrors, and a ring-design field. It has a numerical aperture of 0.1 and has a four times reduction. LLP EUV source was used for this system. A feature size of 100 nm was obtained on this tool [43]. The ETS field size for this system was 24 × 32.5 mm^2^. A micro exposure stepper (MS-13) was developed by Exitech in 2004. It has a numerical aperture of 0.3, five times reduction, and a field size of 0.6 × 0.2 mm^2^ [44]. In 2005, HINA-3 was developed by Nikon which has a NA of 0.3 and a field size of 0.3 × 0.5 mm^2^ [45]. To show the advancement of EUVL, ASML held alpha-demo exposure tools in United States and Europe in 2006. A tin discharge-produced plasma (DPP) light source was used for this tool. This system has six mirrors, four times reduction, and NA of 0.25. The field size of this tool was 26 × 33 mm^2^. 50 nm feature size was achieved by this tool [46]. NXE 3100 was developed in 2010 by ASML. It has a NA of 0.25 which gives a resolution of 28 nm. But the source power of this tool is 10 W which results in low throughput. NXE 3300B was developed in 2013 and has a numerical aperture of 0.33. A resolution of 13 nm can be achieved by this tool and a resolution of 9 nm can be achieved with the help of double patterning technology. The source power of this tool is 55 W and a throughput of 43 wafers/hour can be achieved by this tool [47]. New exposure tools are developed by ASML and ZEISS with high NA such as 0.55 targeting a resolution up to 8 nm [48]. Table 1 shows the development of exposure tools of EUVL with time.

### 3.1. Resist

Mainly two types of resists are used in EUVL: Chemically amplified resist and non-chemically amplified resist. The main challenges for next-generation lithography resist are resolution, sensitivity, and line edge roughness (LER). Along with this outgassing, EUV absorption, and defect density also affect the productivity of the system. Chemically amplified resists (CAR) are commonly used for 248 nm and 193 nm optical lithography. But CAR faced some limitations such as LER, absorption of EUV, and pattern collapsing when used for EUVL. CAR is mainly composed of a photoacid generator (PAG) and polymers. A EUV source of a low exposure dose of 10 mJ/cm^2^ requires high sensitivity and high PAG loading. A 55–100-nm-thick resist is required to control the ratio aspects for the 32 nm node. High etch resistance is required for a thin resist during the transformation of the pattern.

Outgassing can disturb the system and can reduce the lifetime of the optical tools. Outgassing is also enhanced by the high vacuum environment which is compulsory for the EUVL process. According to ITRS, the outgassing rate is 5 × 10^3^ molecules/cm^2^. Non-ionic PAGs and resist have high activation energy that can be used to reduce outgassing. LER is a big challenge not only for EUVL but for all the next-generation lithography techniques. The energy of a photon at 248 nm is 5 eV and at 193 nm, it is 6 eV. While the energy of a photon at 13 nm is 93 eV. This increased energy per photon results in the shot noise and LER. Ultrathin resist of a single layer over a hard mask can be used to control LER. It is also observed that small PAG size and high PAG concentration can reduce the LER [50].

Absorption of EUV light differs in different resists. It is dependent on the atomic absorption in a molecule. Resist materials can be made from the atoms of carbon, hydrogen, and silicon to reduce absorption. Atoms of boron in EUVL resist can also reduce the absorption and enhance the etch resistance [51,52]. The optical density of the resist base was approximately 4.0 μm^−1^ for the transmittances of 67.0% and 44.9% corresponding to the resist thickness of 100 nm and 200 nm respectively. An imaging layer of 120 ± 15 nm thick was suggested. It is commonly assumed that low-energy electrons (LEEs) generated in the resist materials by EUV photons are mostly responsible for the solubility switch that leads to nanopattern formation. Recently, a paper was published which described the role of low energy electrons in a resist made up of tin. It was observed that even electrons with energy as low as 1.2 eV can cause the resists to chemically alter significantly [53]. The knowledge gathered from this study is very helpful in understanding how inorganic EUV resists function in lithographic applications.

#### Advancements in Resist Materials

Results on EUVL resist were presented at the SPIE Advanced Lithography conference in 2007. The parameters required for industrial application of resist are shown in Table 2. An XP6627 CAR was used for obtaining1:1 line: space feature resolution at a 25 nm node. This system had a lower value of 2.7 nm 3σ LER. After these results, it was expected that EUVL CAR can be extended to 22 nm node. While other CAR made up of non-ionic PAGs and polymers can be extended to sub-50 nm resolution. The line: space feature of 1:1 at 50 nm was observed and 1:4 was observed at 20 nm. The sensitivity and LER of molecular resist were improved by 2007 for EUVL. An exposure dose of 12 mJ/cm^2^ with LER 3.1 nm can be used to print the sub-45 nm patterns. A new tool of exposure dose 40 mJ/cm^2^ has been introduced by ASML that reduced the challenge for EUVL resist resolution at 13 nm node. Flood exposure-assisted chemical gradient enhancement technology (FACET) was introduced by Nagahara et al. to improve the roughness, resolution, and sensitivity of the resist [54].

Yamamoto et al. [57] added a metal sensitizer to improve the sensitivity of CAR. The sensitivity was improved due to higher acid yield and an increase in the efficiency of the electrons was obtained. With this, a 43% improvement in sensitivity was achieved. It was also helpful in reducing the LER. A multiscale model was given by Lee et al. [58] which gave an internal look at the various chemical reactions such as diffusion, deprotection, etc., taking place in CAR during the formation of the structure. Polymer loss and LER performance can also be predicted with this multiscale model. Eleven acid amplifiers (AA) were developed and evaluated by Brainard et al. [59] and acid amplifier breaks down when it comes in contact with an acid and produces more acid. ESCAP photoresist was used by Brainard to study the effects of AA. Fluorinated sulfonic acids were produced by the AA. It helped to improve the resolution, LER, and sensitivity of the EUVL resist. Kudo et al. [60] made a pattern of 25 nm node with the help of Noria derivatives synthesized by them. He used an exposure dose of less than 10 mJ/cm^2^. A negative tone CAR was developed by Kulshrestha et al. [61]. Noria molecule was synthesized having oxetane crosslinking moieties. These modifications improved the sensitivity, LER, and swelling. A 1:1 line: space pattern at 20 nm node was created with 3.2 nm LER. A study was made on the impact of molecular weight and processing parameters on the functionality of the resist [62].

### 3.2. Mask

EUVL masks are based upon the technologies of EUVL blank mask preparation and fabrication of masks from raw material. A substrate with a lower coefficient of thermal expansion is required for mask fabrication. The process of mask fabrication from raw material has the following steps: Generation of pattern, pattern transfer, control of quality and defect. The reflectance of the EUV mask is 65–70%. A EUV mask pilot line was established by Intel for the implementation of EUV technology. To control the out-of-plane displacement (OPD), the substrate for the EUV mask must be even. In the 1990s, the expected implementation for EUVL was 100 nm node, but nowadays it is expected to expand beyond 32 nm and 22 nm nodes. A multilayer reflector was etched at about 100 nm to get higher resolution by EUVL by using phase shift mask technology [63,64,65,66]. Zero defect greater than 80 nm polystyrene latex (PSL) equivalent size was required initially at 100 nm node. The defect size for the 32 nm node is 26 nm PSL equivalent size. For 32 nm node, the flatness of 32 nm peak-to-valley (p-v) is required on the front and back for proper contact with the electrostatic chuck. By 2007, a 50 nm p-v flatness goal was expected. A substrate with 120 nm p-v on the front side and 198 nm p-v on the back side gives the best-integrated performance. The thermophoretic protection method was used to overcome the problem of making a non-pellicle EUV mask. Argon gas at 50–1600 mTorr pressure, 65–300 nm particle size, and 2–15 K/cm temperature gradient was used for robust thermophoretic protection [67]. Inspection of multilayer defects is carried out by the non-actinic method and sub-100 nm mask defect detection is done by actinic [68]. The method generally used for inspection of mask blank is multibeam confocal inspection which is a non-actinic method [69,70]. Temperature is also an important factor during mask fabrication. A temperature less than 150 °C is required to control changes in the properties and damage. Absorption of EUV light is also a challenge in mask fabrication. The absorber should have high reflectance and extinction coefficient to decrease the influence of off-axis incidence. In the early days of EUVL, a chromium-based absorber was used which was adopted from binary optical masks [71]. Proximity X-ray lithography also proved helpful for selecting the absorber material. TaBN and TaSi materials were taken from X-ray lithography.

#### Advancement in Mask

EUV masks for 100 nm nodes were required before 2000. The specification for the defect was 0.01/cm^2^. The detection threshold for defects was 50–80 nm. Visible light inspection tools were used for 0.1/cm^2^ for a size greater than 130 nm. The EUVL technology was pushed to 65 nm by 2002. For obtaining a mask blank yield of 60%, the requirement for defect density was 0.0025 defects/cm^2^, this requirement was for both the mask substrate and the multilayer [72]. By this time, there was also progress in the coating technology. Defect levels of 0.05/cm^2^ for defects larger than 90 nm PSL were achieved. EUVL was expected to expand beyond 45 nm or 32 nm node by 2004 [73]. Actinic defect inspection was used for the detection of defects on the blank mask. It detected the defects of width of 70 nm and height of 3.5 nm. The multilayer coating method was also developed at that time. A defect density of 0.055 defects/cm^2^ was detected for particles of size greater than 80 nm PSL equivalent. The lowest defect density detected by this method was 0.005 defects/cm^2^. The dimension of the mask defect should be controlled at 18 nm for 22 nm node. To increase the sensitivity of defect inspection, the actinic detection method in EUVL blank mask was used. Defects of width 50–65 nm and 10–12 nm high were detected with a capture rate of greater than 90% in 2004 [74]. In 2006, photoelectron emission microscopy was used for the detection of defect levels in the EUVL mask. By using this technique, buried defects of 30–50 nm and phase structure of 6 nm high were detected by using the actinic mask blank inspection method [63,75].

**Figure 8:**
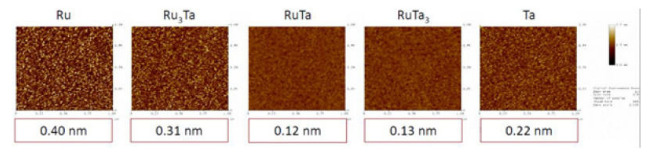
AFM images showing RMS values of Ru and various alloys deposited on 30 nm film [78].

EUVL mask fabrication technology is continuously expanding with the help of electron beam (e-beam). Chemically amplified resists (CAR) enhanced the pattern generation process. The electron beam is not significant to improve CD uniformity and CD mean to target (MTT). A new mask known as the self-mask had proved better over other masks. Multistep etch is used on the absorber layer by this mask. Thermodynamics calculations helped in selecting the chemical system to ensure the functioning of self-mask. Advanced modeling and new characterization techniques made mask development and processing easier [76]. In 2021, a new mass stack model is developed containing multilayers of the Mo/Si which are further covered by Ru layer and an absorber layer of TaBN/TaBo which facilitates the simulation of the performance of the EUVL mask [77]. Ru_4−x_Ta_x_ (x = 1,2,3) is considered the next candidate for EUV low-n mask for which AFM images are shown in Figure 8 [78].

### 3.3. Limitations

EUVL faces the challenge of line edge roughness (LER) and resists resolution. A requirement of a high-vacuum environment limits the use of EUV lithography. It also faces the problem of resist outgassing. Absorption of EUV on resist and effects on resist profile are caused of difficulty in implementing EUV for manufacturing devices. Before EUV is used as an alternative to 193 nm lithography, it has to resolve many critical challenges. To become a cost-effective technology, the power of EUV must be increased at the wafer level. The throughput of currently available EUV source power is not efficient enough. To integrate functional ICs, the defect density on reflective masks needs to be scaled down. The generation and transfer of EUVL mask pattern is a critical challenge. The use of non-pellicle masks is also challenging. Non-pellicle masks are getting replaced by movable pellicle and thermophoretic protection. To get a higher resolution, productivity, and to reduce the LER, resist improvement is required [79]. Much progress is required to make this technique practical.

## 4. E-Beam Lithography

In the development of optical lithography, the major issue is the improvement in resolution. EUVL has difficulties such as resist outgassing and LER. Absorption of EUV in resist material limits the implementation of EUVL. Due to miniaturization and improvement in performance, feature size of semiconductor devices is continuously shrinking. Electron beam lithography (EBL) is introduced because of its capability of highest practical resolution due to small wavelength of electrons (less than 0.1 nm for 10–50 keV electrons) and its resolution is set by resolution of resists. The diameter of the electron beam used should be smaller than the final structure for higher resolution. For nanoscale patterning, electron beam lithography is a useful technique that was discovered in the early 1960s. High-performance 36 nm gate length CMOS devices are successfully fabricated by e-beam lithography. A 100 nm T-gate GaAs PHEMT and single electron tunneling (SET) devices [9] with high-density low-power memory, fully scaled 0.5 µm CMOS devices, and 0.25 µm NMOS and PMOS devices are successfully fabricated with EBL. Devices such as cryo-electric and optical devices are also developed by EBL by coupling with other lithographic techniques [80]. By using 150 kV high accelerated voltage, high-aspect nano-groove is fabricated in thick-film resists by this technique successfully [81].

It is a technique in which patterns are written by using a finely focused beam of electrons (having a diameter in sub-micrometer range) in the thin films of electron-sensitive materials. It can print very complex patterns with great accuracy directly on wafers. EBL is similar to scanning electron microscopy (SEM) and photomasks which are used for optical lithography and nanoimprint lithography are fabricated by this technique. EBL is a technique with minimum feature size, having great accuracy in registration of one pattern with another over a small wafer area, and defect density lower than any other technique and there is no need for masks in this technique. Here resists of geometries in micron and sub-micron range are generated, and depth of focus is greater than any other technique. It has higher resolution, high density, high sensitivity and reliability than EUVL.

**Figure 9:**
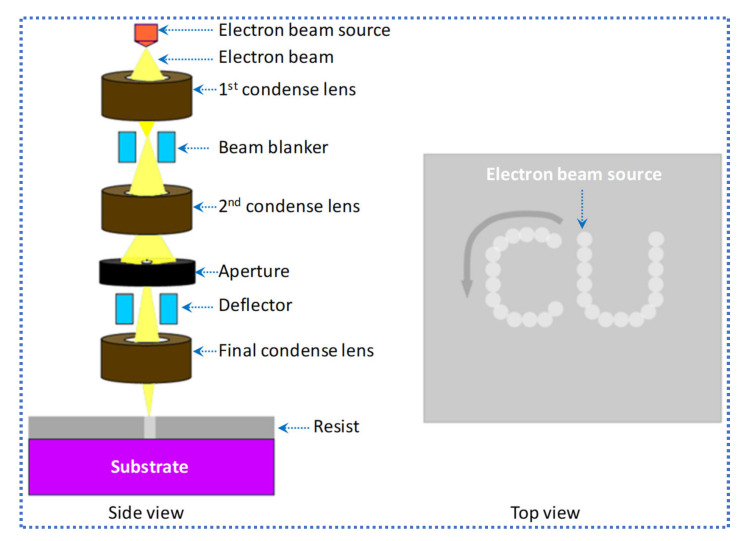
Schematic diagram of EBL system.

In top-down nanofabrication, EBL is the most widely used direct-writing and high-resolution patterning tool. Modern EBL machines can write the structures of nano-sized on areas up to mm^2^. The principle of working of EBL is very similar to photolithography, in which a resist-coated substrate is scanned by a focused electron beam and its solubility properties are changed according to the energy deposited by a beam of electrons and the developer removed the areas exposed or unexposed according to the resist’s tone. EBL consists of a chamber, electron gun, and a column. Figure 9 shows the EBL system, consisting of all the required parts to focus and scan the electron beam.

The column and chamber are highly vacuumed. All the electron-optical elements that are used to generate a beam of electrons, accelerate it to working voltage, turn on and off, focus, and deflect it as needed by the writing pattern are contained in the column. In the main chamber, a load lock loaded the samples normally, which are placed on interferometric stage for the accuracy in positioning of working piece. The EBL patterned sample is obtained by the following steps: (a) conceptual design, (b) CAD design, (c) conversion and proximity correction, (d) sample preparation, (e) machine calibration, (f) exposure, and (g) development [11].

According to beam shape EBL is classified in two categories i.e., Gaussian beam and shaped beam; Gaussian beam is further classified as raster beam and vector beam. Vector scanning is a time-saving system. In EBL for pattern writing, two schemes are used: direct writing and proximity patterning. Direct writing is better than proximity printing having a range from finely focused gaussian spot to a complex shape, and proximity printing is still under development today.

**Figure 10:**
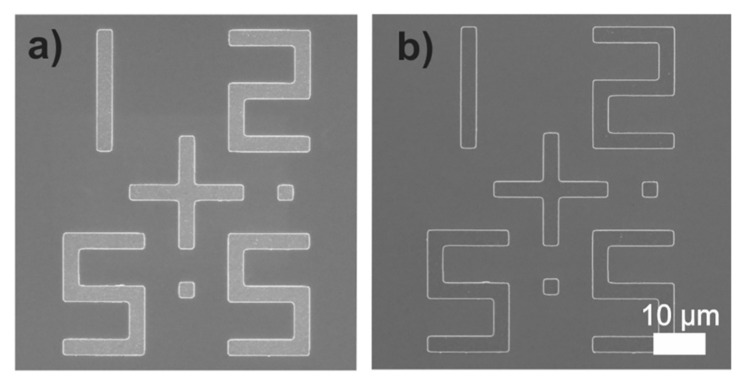
Alignment marks in OIR. SEM images at 5 keV showing Al alignment marks on silicon substrates, (**a**) before and (**b**) after condensing 80-nm-thin nonane ice film. Reprinted with permission from [96]. Copyright 2018, Elsevier.

In order to improve the throughput of e-beam lithography, multiple e-beam direct-write (MEBDW) lithography concepts have been introduced having >10,000 e-beams writing parallelly, with nanometers resolution [82]. In the 1970s, more elaborate machines were developed that exposed larger fields of view up to 5 × 5 mm^2^ with an edge resolution of 0.25 pm. In 1980, many commercial systems were available for direct writing and mask making [83]. Further, MAPPER lithography based on parallel electron-beam writing with high-speed optical data transport was developed. Optical columns can be made with a throughput of 10 wafers per hour by this technique [84]. With a target of 40 wafers per hour throughput Mapper’s 3rd generation platform (FLX) has been established with the use of 650,000 beamlets [85]. IMS (which is a single source with many spots in a single lens field, having 50 kV raster mask writer) [86] and a multibeam wafer writer are also developed to improve the throughput [86]. A massively parallel electron beam direct write (MPEBDW) system is developed for digital fabrication of ICs [87]. MPEBDW is further developed by using active-matrix nanocrystalline silicon (nc-Si) electron emitter arrays. Through miniaturization electron optic column multi- column MPEBDW system development is also being planned due to which a practical level of throughput is achieved for commercial semiconductor manufacturing and future mass production [88]. However, there are some abnormal beams in massively parallel electron beams that affect the throughput [89]. Thus, with further advancements in EBL, focused electron beam induced deposition (FEBID) is introduced which is a direct-write nanofabrication technique [90]. It is a powerful 3D nanomagnetism tool that has the capability of unique fundamental studies of 3D complex geometries and its specialized application is compatible with low throughputs [91,92,93,94]. Zhao et al. developed a 3D EBL which uses ice as resist, due to which steps are reduced and 3D nanostructures are easily fabricated [95]. A picture of the alignment marks attained during organic ice resist (OIR) patterning is shown in Figure 10 [96]. In the past decade, it was reported that maskless EBL has the capability of resolution less than 10 nm [97]. For further improvement in EBL technique, recent developments in processing, tooling, resists, and pattern controlling are separately examined [98].

### 4.1. Resists

PMMA (poly methyl methacrylate) is the most widely used resist in EBL in the very early days. It has a good balance of contrast, sensitivity, and roughness. PMMA can be used as both positive and negative resist in the fabrication process. When it is exposed to an electron beam, it breaks into fragments of lower molecular weight, which are removed by the developer such as methyl isobutyl ketone (MIBK) in propanol, so it behaves as a positive resist and when they are exposed to higher doses, >50–70 C/m^2^ for a layer having thickness 1000 Å, PMMA molecules get crosslinked with each other and form a larger network of molecules which act as resistant to developer solvents such as acetone and behaves as negative tone resist. At the end of 1980s, another positive tone resist i.e., ZEP was introduced because of its better resolution and sensitivity than PMMA. These resists (PMMA, ZEP) belong to the methacrylate family and are easily handled and have simplicity in chemical formation.

New resists of this family are SML or CSAR, which are organic, positive tone resists that possess higher sensitivity, resolution, and etch durability. For further advancements, chemically amplified resists (CARs) are introduced and developed by IBM having better sensitivity and resolution [33]. These are acid reactive polymers and PAG (photo-acid generator) [99]. Recently, a negative tone CAR was developed for EBL consisting of glycidyl methacrylate (GMA), methyl methacrylate (MMA), and triphenyl sulfonium salts methacrylate (TPSMA) [100]. GMA and MMA were polymerized together (GMA-co-MMA) and give sensitivity (300 µC/cm^2^); further (GMA-co-MMA-co-TPSMA) were polymerized (avg. mol. wt. = 23,800 g/mol) and sensitivity of 125 µC/cm^2^ was measured. Some positive tone CARs have also been used such as 40TX [101]. Although CARs have many advantages, they also have some limitations such as photoacid is released upon electron beam irradiation which acts as a catalyst and affects the sensitivity. To overcome this issue some new resists are introduced i.e., n-CARS [102]. A polymer of (4-(methacryloyloxy) phenyl) dimethyl sulfonium triflate (MAPDST) and a radiation-sensitive group (MMA) methyl methacrylate was synthesized (MAPDST-MMA) [103]. It dissolves in methanol and forms a negative tone resist which gives a very high sensitivity. Tada et al. [104] observed that fullerene (C_60_) can behave as a negative tone EBL resist. However, in the last few years, some new fullerene derivative polymers were synthesized which act as resists in EBL. After using many organic resists some inorganic resists were introduced because they have better etch resistance and higher contrast than organic resists. HSQ is the most commonly used inorganic resist belonging to the polyhedral oligo-silsesquioxane family [105]. There are some sensitivity issues and it has a short shelf life, therefore, it becomes difficult to work with it, hence some new inorganic resists were introduced. In 2007, two new resists of negative tone were employed, containing hafnium and zirconium oxide sulfates [106]. These resists are atomically dense and have higher sensitivity and resolution than HSQ [107]. Some water-soluble resists have been introduced to make the processes environment-friendly and less hazardous to humans and also to increase the etch durability. Therefore new resists such as poly (sodium4-styrene sulphonate) (PSS) have been developed which showed 17 times higher etch resistance than PMMA [108]. Recently, engineers at Tufts University, USA introduced the idea of using silk as a resist for nanofabrication. They proved that the protein structure of silk was changed upon EBL irradiation, which can be used as either positive or negative resist. Thus, silk is a new eco-friendly resist that helps in reducing the need for toxic chemicals. Table 3 shows advancement in resist materials and improvement in resolution with EBL.

### 4.2. Limitations

**Figure 11:**
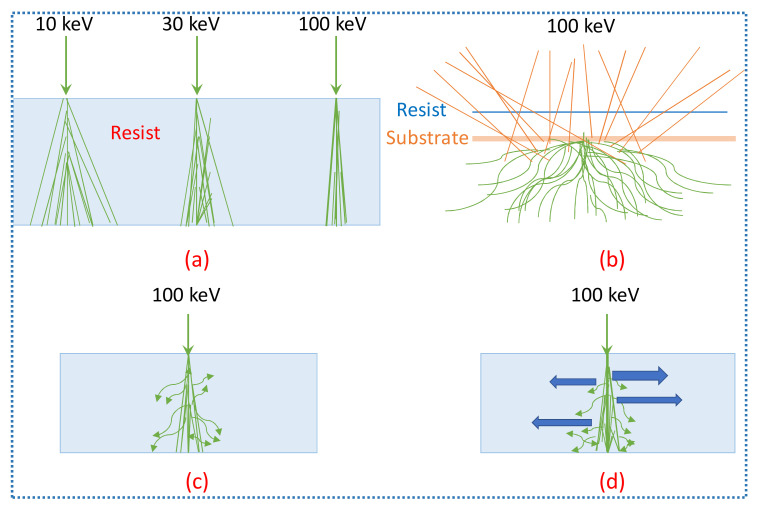
(**a**) Forward scattering, (**b**) backscattering, (**c**) secondary electrons, (**d**) volume plasmons.

The central problem of this technique is throughput. It has very low throughput because it is a serial process, it is very slow and it takes a lot of time i.e., it takes several hours to produce patterns whereas in optical lithography the patterns are produced on wafers within a few minutes. Electrical and magnetic noise can cause perturbations with the electron beams. Therefore, there is a need for proper shielding or there should be a proper separation between beam-column and computer monitor, pumps, and transformers [112]. The intensity of electron beam and sensitivity of resist are also limited. For higher current beams, trajectory displacement and chromatic aberration are the major issues in focusing the electron beam. Due to the point spread function (PSF), the exposure region of resist spreads away from the beam spot center. Some of the effects observed are shown in Figure 11.

Scattering is one of the major limitations of EBL technique. Resolution is also limited by secondary electrons having energy in the 2 to 50 eV range [112]. One of the observed effects is volume plasmons as shown in Figure 11d in which some charge waves called volume plasmons move in resist causes alteration in chemical bonds because of the electrons of very high energy passing through the resist [113]. These limitations observed in EBL reduce its use. Some new next-generation lithographic techniques such as X-rays, ion-beam lithography, etc., are developed to overcome these limitations.

## 5. X-ray Lithography

**Figure 12:**
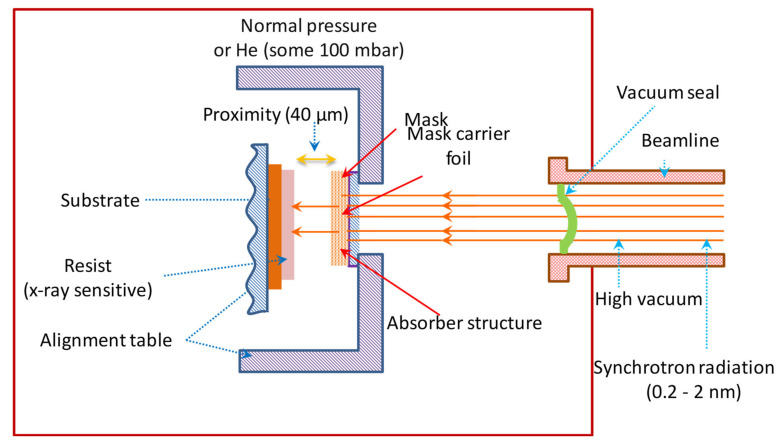
Schematic view of the X-ray lithography system.

X-ray lithography is one of the modern forms of lithography having many advantages over E-beam lithography and photolithography. One advantage over photolithography is that X-rays have shorter wavelengths (of the order of 0.1–10 nm) than UV light used in photolithography due to which it has higher resolution and there are no diffraction limits. Due to smaller wavelengths, the patterns are highly precise. It is less expensive and the throughput is higher than EBL. By using XRL, the size of transistors is reduced to 17 nm from 130 nm. It is a fast technique with a high aspect ratio. All the scattering-related problems faced in EBL are eliminated in XRL because the index of refraction of X-rays is almost unity, reducing the reflection of these rays. The main application of XRL is the successful and well-established mask technology consisting of a thin layer of mask of low Z-material. Comb-drive microstructures are fabricated by XRL with the use of polyimide-Au X-ray mask [114]. For microstructure fabrication, XRL is a versatile tool [115]. Various micro scale devices such as 64 Mb dynamic random access memories (DRAMs) are fabricated by IBM using XRL. 1 Gb DRAM test site is fabricated by Mitsubishi. CMOS logic devices, with a 12 Kb SRAM and 48 × 48 bit multiplier are fabricated by NTT with 0.2 µm features. Motorola fabricated the CMOS logic devices (which include a fully functional 1 Mb static RAM) with feature sizes of 0.375 µm. In recent years, gratings are also fabricated by XRL [116] because most of the fields such as medical, tomography, biology, etc., focus on gratings [117]. XRL has the potential to fabricate about 1 µm linewidth gratings [115]. Recently, a nanoscale reference grating with a deviation of below ±0.5 nm and a mean pitch of 0.01 nm is fabricated by Deng et al. [118]. H. Smith and Spears at MIT were the first who proposed X-ray lithography [119]. X-ray systems were developed by Bell laboratories by using Pd stationary targets [120] and many other companies in the U.S. followed the approach of Pd targets. In XRL, geometric patterns are transferred to the surface of wafer from a mask by using a collimated X-ray beam. The main components of XRL system are: a high-power X-ray source, beamline, a mask, resist, and exposure tools. All the important components are shown in Figure 12.

The main factors which determine the performance of any lithographic technique are resolution, critical dimension control, and overlay accuracy. For any XRL system, the basic requirement is a highly reliable and bright X-ray source. In earlier days, X-ray tubes were used as X-ray radiation sources but these were not suitable for lab applications because they have large focus diameter and poor intensity. For an effective technology, high-power sources are required. To fulfill this requirement, plasma sources are introduced. The radiation intensity of these sources is higher and has a relatively small focus than X-ray tubes. In the Naval research lab, many experiments were performed in the 70 s in which the usefulness of plasma-generated sources was introduced for lithographic application. Generally, two types of plasma sources are used i.e., laser-induced plasma sources and discharge-plasma sources. Laser plasma sources have relatively lower radiation intensity than discharge plasma sources and also at the time, there is no availability of low-cost, high-power lasers, which led to the use of discharge plasma sources in a large amount. Due to some limitations of plasma sources such as electrode erosion and also plasma sources damage the mask and wafer, these sources are no longer used as X-ray radiation sources.

Over the past 15 years, several signs of progress have been made on all the components of XRL (i.e., source, aligner, mask, resist). By the early 1980s, XRL has been under development. Many research groups and industries such as IBM, Motorola, Lucent in USA, and some of the industries of Japan (Fujitsu, Hitachi, NEC, NTT) have carried out a lot of work to develop XRL and several universities such as MIT and University of Wisconsin have been doing some additional work to develop XRL. Recent improvements in X-ray sources introduced synchrotron radiation sources which are the brightest sources of X-rays produced by storage rings having high intensity and high throughput and for further advancements, compact storage rings are introduced. In 1980, at IBM Yorktown, storage ring X-ray sources were used in the XRL technique. In storage rings, electrons emit synchrotron radiation, which is strongly collimated in a forward direction having a high depth of focus (~10 µm) and high intensity (up to 100 mW/cm^2^). In 1993, Oxford instrument [121] built the storage ring Helios1 [122] which has been operating at IBM’s Advanced Lithographic Facility (ALF) in East Fishkill, NY, USA [121]. Further advancements were made in 1996, the system with higher beam currents was upgraded and it stored current of over 500 mA. Now the performance of beamline and storage rings is quite appropriate for XRL at 0.13 µm and below. Steppers are used in XRL for increasing the throughput. Further improvement in XRL is done by using a stepped attenuator of X-ray beam [123]. Special masks of silicon carbide with unique properties are needed for this technique which is thinner than optical lithographic masks. There is a separation of a few microns between mask and wafer. The resists used in X-rays are thinner and are more sensitive than photoresists (~2 µm). Several companies such as IBM, Fujitsu, NEC, NTT, Hitachi, Toshiba, etc., currently support the XRL development [121].

### 5.1. Resists

X-ray lithography requires a radiation-sensitive material called resist on which patterns are transferred from a mask. Different X-ray resists are shown in Table 4. As X-rays are of shorter wavelengths, it requires a single-layer resist material with very high resolution and are highly sensitive. The basic requirements for X-ray resists are: it should possess a good submicron resolution (better than 0.1 µm) with adequate thickness, good thermal uniformity, and thermal stability, have appropriate sensitivity to incident X-ray flux (i.e., better than 100 mJ/cm^2^), exposure time is about 2 s per step field assuming 250 mW/cm^2^ for high throughput, dissolution ratio and contrast should be ≥10, and it should have low defects [124]. The exposure of resists in E-beam and XRL are very similar processes and there is a correlation between sensitivities of X-ray and e-beam resists. In both cases, the energy of incident particles is much greater than the required energy to break a chemical bond. When an X-ray photon enters the resist, it produces many secondary electrons having very high energy than the incident photon, indicating a strong interaction with resist material. These secondary electrons define the limit of resolution of an X-ray resist and are responsible for all chemical changes in resist [125,126]. Practically, all the resist materials are thin films of organic polymers. PMMA is the best-known resist for XRL because it has the highest resolution among all the resists [127]. It is an e-beam resist and was discovered by Haller et al. [128], but it has some limitations also i.e., it has a very low sensitivity of about 1000 mJ/cm^2^ and its etching stability is also poor. Many attempts were made to improve its parameters but all attempts failed, due to which some new resists were introduced. IBM research XRL program has used resists that are compatible with the semiconductor process. IBM novolac positive resist gives a linewidth control of 0.007 µm in a 1-µm thick resist layer and the resists required a dose of about 700 J/cm^3^ [129]. The technological stability of standard novolac-based resists is good, making it a demanding one for future lithographic techniques. Some optical resists i.e., novolac/diazotype show better results in terms of resolution and stability. Some single-layer resists such as HPR204 and HUNT WX 214 show the best results with high resolution and good pattern fidelity is achieved with these resists. To further improve the sensitivity of novolac resists, the “chemical amplification” concept is introduced [127]. So, it is concluded that commonly used resists in XRL are chemically amplified due to their higher sensitivity. Some DUV and e-beam resists are also used in XRL such as TDUR series (Tokyo Ohka), SAL series (Shipley). Due to the absence of backscattering effects, XRL should be able to give equally high resolution with negative type resists. Several experiments were performed with two negative-type resists i.e., KMNR and epoxidized poly-butadiene (EPB) and it was observed that the sensitivity of EPB is about 100 times that of PMMA, but due to low contrast, sharp patterns are not produced. While KMNR is about seven times more sensitive than PMMA and has good contrast and better resolution. In XRL chain, scission type resists have relatively low sensitivity (100–200 mJ/cm^2^ at 8 Å), so crosslinking resists are used with a sensitivity of about (10 mJ/cm^2^ at 4.37 Å). Recently, some positive and negative resists [130] with a sensitivity of about 50 mJ/cm^2^ at 8 Å have been realized [119]. In low-Z materials, there is a deposition of considerable amount of energy in resist through the Auger process. Chlorine-doped negative resists are the most sensitive X-ray resists in practical use today. Due to the presence of “Cl” in these resists the resist absorption increases by a factor of 10 than PMMA. Further improvements in sensitivity introduced a highly sensitive resist DCPA. Resolution is the major resist-related issue, so there is a need to develop XRL resists with higher sensitivity and higher resolution and some of the commercially available resists are Apex-E, UVII-HS, UV-4, TDUR-N900, and SAL601 [131].

### 5.2. Aligners

**Figure 13:**
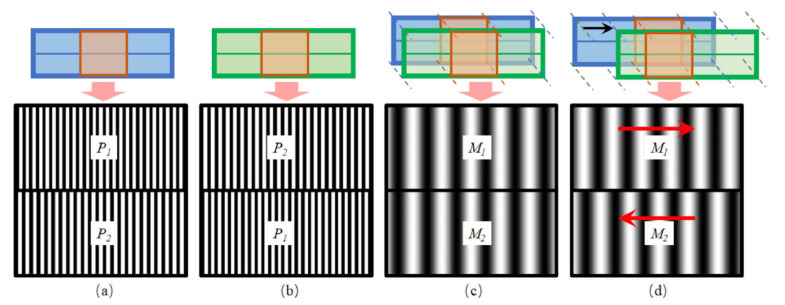
(**a**) Alignment of wafer marks, (**b**) alignment marks of mask, (**c**) Fringes of Moire in perfectly aligned wafer and mask and (**d**) Fringes of Moire in imperfectly aligned mask and wafer. Reprinted with permission from [136]. Copyright 2021, Elsevier.

For the fabrication of electronic circuits several steps are required, for which different patterns are produced on wafers at different times and these patterns should be properly overlayed with several aligners/steppers. In XRL, the aligners can be divided into two systems: one is an alignment measurement system and the other is a mechanical positioning system. Figure 13 depicts the alignment marks for the wafer mask, perfect alignment of wafer to mask and misalignment of a wafer to mask [136]. Some aligners are based on the linear Fresnel Zone Plate scheme. X-ray aligners are more advantageous than optical aligners because lenses are not used in these aligners as in optical aligners. Stepper manufacturers investigated some new approaches to improve magnification. SVGL is one of the approaches which is based on the system on which Chen and Silverman worked earlier [121]. The first X-ray mask aligners are commercially available for laboratory applications but are suitable for X-ray tubes or plasma sources only. IBM (Brookhaven) and cooperating group in Berlin (Suss, seimen, IMT) built the prototypes of X-ray steppers, which are operating with synchrotron radiation but the speed of these prototypes (MAXI) is very low. MAXIII or XRS200 is the new generation prototypes under construction. Several companies such as Canon, SAL, SVGL, NTT have developed X-ray steppers and the tools are commercially available. XS-1 is a stepper being developed by the ASET XRL group.

Some aligners simultaneously measure the alignment and expose the wafer, have been recently developed by Perkin-Elmer and NEC [137] which are based on the scheme of linear Fresnel zone plate and were developed at Thomson CSF and NTT [129,138]. Several other companies also developed the additional aligners. SVGL tool is the most advanced aligner in ALF, with full standard mechanical interface (SMIF) handling of masks and wafers, it has been demonstrated as a production-worthy tool. The overlay performance is nearly 35–40 nm by using the global alignment and measuring overlay errors. The alignment accuracy of 15–19 nm has been reported by Canon stepper which was installed at Mitsubishi while stability of 17 nm has been reported by a SAL stepper which was installed at the University of Wisconsin. Recently, maskless XRL is introduced which uses a double-Fresnel Zone Plate lens to write features of about 15 nm [139]. Still today, there is a need for great efforts to improve the alignment systems.

### 5.3. Limitations

At present, the most critical issue in XRL is the mask technology and defect elimination tools [124]. As the masks used in XRL are very thin they can be deformed or bent easily. Another issue with X-ray masks is vibrations. Vibrations can move the masks from their alignment and if masks are not properly aligned with wafers, then the transistors on wafer do not work properly. X-ray masks are made by using e-beam lithography and it is a very slow process [121]. One of the main issues is that distortions are caused due to mask heating. Unlike photolithography, lens is not used in XRL to focus the X-rays on wafers, the patterns on wafers are of the same size as that on mask and to produce small patterns on wafers the patterns on mask should be made small. Another disadvantage is that, although storage rings are powerful sources of XRL, but these machines have a huge size and large physical dimensions [124]. One of the biggest remaining issues is the development of XRL resists with higher resolution and sensitivity, and to overcome these limitations XIL (X-ray interference lithography) is replaced by XRL, which is a promising tool for future nanofabrication and some new and more advanced lithographic techniques such as nanoimprint lithography, ion beam lithography, etc., are introduced [140].

## 6. Ion Beam Lithography

The diffraction constraints that occurred in optical lithography have been resolved by NGLs such as EUV, X-ray, and ion beam lithography. E-beams spread when entering into the resist material, while ion beam travels in a straight line which causes less spread of beam and makes it better for fabricating high aspect ratio 3D structures. The penetration depth of the ion beam is well defined and can be changed by varying the ion energy. Large-scale patterns can be generated using ion beam projection with the help of nanoimprinting technology. A 0.9 µm Si n-type metal oxide semiconductor (NMOS) [141], GaAs metal-semiconductor field-effect transistor (MOSFET) [142] of 0.8 µm and 0.15 µm gate, MEMS devices, photonic, plasmonic structures, and magnetic structures are successfully fabricated using IBL. Any material can be machined using FIB by the surface erosion method. FIB has a great implementation in micro-technology and metrology. Nanoimprinting which has been included in NGLs for the first time in 2003 by ITRS depends on the quality of stamps that can be fabricated with the help of FIB. Ion beam lithography has three techniques (i) focused ion beam (FIB), (ii) proton beam writing (p-beam writing), and (iii) ion projection lithography (IPL) which are discussed below.

**Figure 14:**
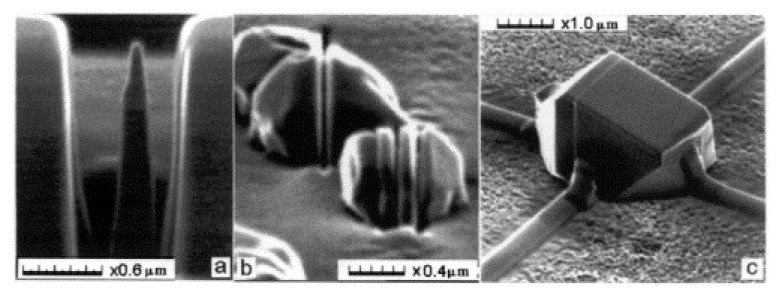
Structure fabricated using FIB; (**a**) nanotip in 1-mm thick ta-C film, (**b**) nanoscale trenches in diamond microcrystallites, and (**c**) tungsten contacts. Reprinted with permission from [143]. Copyright 2001, Elsevier.

**Focused ion beam (FIB)**—FIB is the most developed technique among all the ion beam lithography techniques. It was developed in the later 1970s. The instruments of this technique became commercially available approximately 10 years later. It is a direct-write process in which a beam of slow heavy ions is used to sputter the atoms or for modification of the surface patterns. Using FIB virtual patterns can be produced in any material which makes FIB different from all other nanotechnologies. But this process is slower than other processes. Gas-assisted etching (GAE) has significantly enhanced the etching rate of the process. Recently, poly-methyl methacrylate (PMMA) with FIB sputtering has enhanced the surface sputtering rate by several orders in magnitude. 3D nanofabrication has been developed using FIB chemical vapor deposition (CVD). Bulk diamond are patterned using FIB as shown in Figure 14 [143], author used a focused Ga-ion beam of 50 keV to fabricate sub-100 nm pattern. Carbon nanopillars are grown using FIBCVD [144]. Ordered nanochannels are fabricated in alumina using FIB.

There is an advancement in ion sources of FIB in the past several years. Liquid metal ion source (LMIS) is used in commercial microscopes based on FIB. FIB technique based on Ga^+^ LMIS is commonly used for its stability, long lifetime, and rapid machining due to its high mass ions. LMIS source has a brightness of 10^6^ Am^−2^sr^−1^eV^−1^. Recently a new tool for the bit milling of 2D and 3D nanostructure is developed with the help of FIB [145]. But nowadays, gas field ionization sources (GFIS) having He^+^ ions have been developed. These sources are capable of achieving etch lines of sub-10 nm. Along with this, ICP (inductively coupled plasma) and LMAISs (liquid metal alloy ion sources) have been developed which have capabilities for wide milling rates and for doping at nm scale respectively. A promising ion source namely LoTIS (laser-cooled low-temperature ion sources) is developed recently which has a brightness of 2 × 10^7^ Am^−2^sr^−1^eV^−1^.

**Figure 15:**
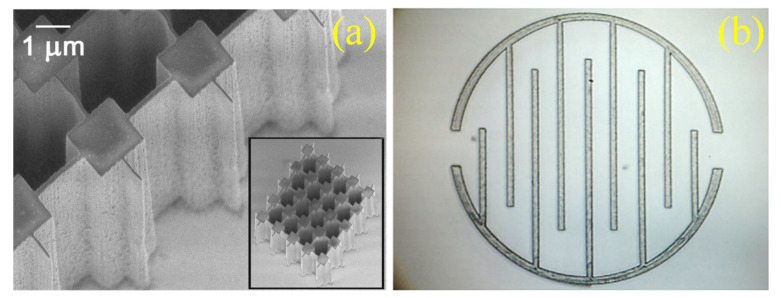
(**a**) Microscope of 2 × 2 μm^2^ pillars fabricated in SU8 negative resist using p-beam writing with energy of 1 MeV. Reprinted with permission from [146]. Copyright 2003 AIP Publishing. (**b**) Fabrication of micro walls in PDMS. Reprinted with permission from [147].

**Proton beam writing**—This is a maskless lithography technique. A beam of focused protons of energy of several MeV is used to write directly on the resist. The high energy of the proton results in higher penetration into the resist material. The removal rate of approximately 10⁶ µm^3^ per nC of protons makes this process million times more efficient than the FIB process for 3D patterning. Recently, it has been observed that proton writing is very effective in the fabrication of multi-level structures. P-writing is effective in fabricating a high aspect ratio structure in silicon when silicon is used as a negative resist. Figure 15a shows capabilities of the technique for fabricating high aspect ratio walls of 60 and 120 nm width with sub-3 nm edge smoothness using SU8 negative resist [146] and Figure 15b shows micro walls created in PDMS using PBW [147]. The author reported that the secondary electron has low energy resulting in a minimal proximity effect. A new tool to determine the beam intensity is developed by ISOLDE [148].

The limitation of p-writing is that it is an underdevelopment technique and commercially no instruments are available yet. Another difficulty is faced during the focusing of p-beam of MeV ions at a 100 nm scale. Recently, beam focusing difficulty has been resolved by the compact magnetic quadrupole lens system. The first prototype for p-beam has been developed by CIBA (Centre for ion beam applications). Multiple lens arrays are fabricated using p-writing and FIB. For the study of patterned porous silicon, the CIBA group in Singapore has developed p-beam micromachining of semiconductors [149].

**Ion projection lithography (IPL)**—IPL uses the ions such as protons, H^2+^, He^+^, Ar^+^, etc., in the energy range of 50–150 keV. It has advantages over FIB and p-writing for cost-effective mass production. IMS (Ion Microfabrication System GmbH, in Vienna) pioneers the IPL. A beam of ions is illuminated on the large area of stencil mask. Electrostatic lenses are used to project the transmitted beam in a European consortium. IMS has patterned at 50 nm resolution in parts of the exposure field. IMS has succeeded in patterning over the full field of 12.5 × 12.5 sq mm at a 75 nm scale using a single beam exposure system [150]. IMS has also developed a new variety of IPL which is called ion projection direct structuring (IPDS). Magnetic anisotropy of the storage system is enhanced by using ions. This system succeeded in patterning at sub-109 nm scale at extremely high densities. A new device named PROFIB (projection focused ion multi-beam) has been developed by IMS with 200× reduction optics for future technology.

The first IPL tool developed was the ion projection machine (IPLM). It was developed in 1988 and used for demagnifying ion optics of dimensions 5× or 10× for reducing open stencil masks at sub-100 nm scale. The second IPL tool was developed by IMS in 1990. It was named ALPHA-10X. After some modifications, the field was increased to 8 × 8 mm^2^ in 1991. Then a new tool ALG-1000 was developed in 1992. It can expose a field of 20 × 20 mm^2^ at 3× reduction. Process development tool (PDT) was developed in 1997, which can expose a field of 12.5 × 12.5 mm^2^ by IMS. It can achieve resolution up to 50 nm.

### 6.1. Resist

To overcome the limitations of single-layer resist, multilayer resists systems are becoming popular. Top layers are made thin to reduce the resist contrast. It is necessary to use thin top layers in ion beam lithography, particularly in focused ion beam lithography in which heavy ions of Ga or Si are produced to get the short stopping distance into the resist material. Recently two layers resist system is commonly used in which the top layer is imaged and after its development, the image is transferred to the thick bottom layer. The thickness of bottom layer is 2–3 µm. The image is transferred to the second layer by reactive ion etching (RIE). The top layer is resistant to oxygen RIE.

#### 6.1.1. Organic Resist for FIB

**Figure 16:**
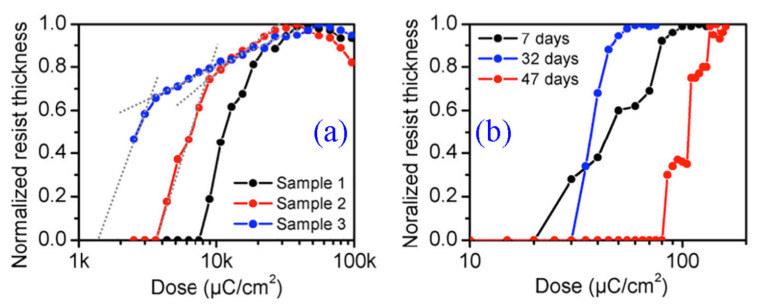
(**a**) EBL contrast curve of different samples at different development time. (**b**) FHIB contrast curve. Reprinted with permission from [153], Copyright 2018, Elsevier.

An organic resist PMMA is a positive tone resist. In the 1970s, PMMA of average molecular weight of 1.85 × 10^5^ g/mol was patterned with helium ion of dose approx. 17 µC/cm at a feature size of 2.7 µm. Shi et al. [151] took the PMMA of molecular weight 4.95 × 10^5^ g/mol and revealed that PMMA acts as a negative resist at an increased dose. The results showed that PMMA is more sensitive to HIB rather than an e-beam. The dose for HIBL was observed to be ~2 µC/cm^2^ and the dose for EBL was ~120 µC/cm^2^ which shows that PMMA is 60 times more sensitive toward HIB than e-beam. While for negative tone PMMA resists, the dose of sensitivity was observed to be 68 µC/cm^2^ [152]. Figure 16a illustrates the EBL contrast curve depicting the effect of post baking and delay in development. Sample 1 was post baked and developed after the exposure, sample 2 was baked after exposure and developed after 24 h and sample 3 was post baked and developed after 24 h from exposure. The delay in post baking and development increased the sensitivity of the resist. Figure 16b depicts the contrast curve of the same resist after storing the samples for 7, 32, and 47 days at room temperature and exposed with helium ion beam. The results showed that the resist was two times more sensitive to the FHIB than the EBL [153].

Hydrogen silsesquioxane (HSQ) is an inorganic negative tone resist. Sidorkin et al. [154] Studied the isolated dot patterns on HSQ film scanned with helium ions beam. At HSQ films of thickness 5 and 55 nm, isolated dot patterns were observed at a pitch of 98 nm. At 5 nm thickness, a dot pattern of diameter of 6 nm was observed. While at 55 nm thickness, a dot pattern of a diameter of 14 nm was observed. This sensitivity of HSQ was observed with a helium dose of 31 ± 3 µC/cm^2^. This dose is 4.4 times less than the dose for EBL. Winston and their team fabricated 20 nm and 10 nm structures in HSQ with a line dose of 0.0834 nC/cm. There is another Hafnium-based inorganic resist studied by Luo et al. [155]. When HafSOx is exposed to helium ions, the feature size was patterned at a dose of ~4 µC/cm^2^. The same sensitivity for e-beam was observed at a dose of ~420 µC/cm^2^. Another inorganic resist is alumina based when exposed to helium ions beam patterned isolated lines of 5 nm at 20 nm pitch. With FHIB of 30 keV, various pitches of 20 nm, 40 nm, and 64 nm were established with doses varying between 200 and 700 µC/cm^2^. But the resist has limitations due to the lifetime of resist solution.

After this resist, new resists of the organic-inorganic hybrid were developed. MAPDSA-MAPDST is such a resist which can pattern 20 nm feature size with a helium ions dose of 6 µC/cm^2^. The LER value was (1.27 ± 0.31) and the sensitivity was 7.2 µC/cm^2^. Later, another organic-inorganic hybrid n-CAR was developed named MAPDST-co-ADSM. When this resist is exposed to helium ions, sub-15 nm patterns were observed with a dose of nearly 50 µC/cm^2^. Single-pixel exposure was used to obtain a line pattern of 10 nm with a dose of nearly 50.48 pC/cm.

#### 6.1.2. Positive Resist for Proton Beam

Uchiya et al. reported that PMMA of 5 µm thickness is exposed by a proton beam of 1.7 MeV protons, and holes nearly of 130 nm wide were observed on the PMMA surface with an ion dose of 3.8 × 10^3^ ions/cm^2^ [156]. The PMMA sample is developed in isobutyl ketone (MIBK)/IPA in a ratio of 1:3 for 5 min and rinsed with ethanol followed by drying. Then, Menzel et al. [157] showed that PMMA exposed by a proton beam of energy 2.25 MeV can clear the exposed area with a dose between 2.5 × 10^1^^4^ and 3 × 10^1^^4^ ions/cm^2^. Andrea et al. [158] reported that 3D structures can be machined on PMMA bulk at LIPSION accelerator facility with the help of ion energy. Erps et al. [159] reported that micro-optical and micro-mechanical components can be fabricated in PMMA resist up to a thickness of 2 mm using a deep proton beam of energy 16.5 MeV.

Larisch et al. [160] prepared 4 µm agar film and exposed it with a proton beam of energy 2.25 MeV protons. Structures of 15 µm were fabricated with a dose of 3 × 10^1^^4^ ions/cm^2^. They used agar for biological patterning.

#### 6.1.3. Resist for Proton Beam

The first negative resist amicable with proton beam writing was SU-8. In 2003, features of sub-100 nm were revealed at CIBA. SU-8 of 10 µm thickness was exposed by proton beams of 1 MeV protons with a dose of 1.9 × 10^13^ ions/cm^2^. They were successful in achieving wide lines of 60 nm [146]. In 2009, 3D-electric micro filters with pillar arrays of high aspect ratio of height approx. 15 µm and a diameter of ~1 µm were observed by Furuta et al. [161]. SU-8 resist of thickness 15 µm was exposed to proton beam of energy 1.7 MeV to fabricate pillars with ion dose of 6.3 × 10^13^ ions/cm^2^. SU-8 resists exposed to 2.25 MeV protons at an ion dose of 1.8–30.6 × 10^12^ ions/cm^2^, grayscale structures were fabricated by Menzel et al. [162].

HSQ is a negative resist developed in 2.38% tetramethyl ammonium hydroxide for PBW. HSQ provides high resolution for this technology. In 2010, HSQ of thickness 20 nm was exposed to 10 keV H^3+^ at the dose of 7.5 × 10^13^ ions/cm^2^, and structures of 20 nm width were fabricated by Van Delft et al. [163].

TADEP is a chemically amplified negative resist. In 2008, Chatzichristudi et al. [164] discussed the fabrication of structures of size 1.5 µm using 2 MeV protons at a dose of 1.9 × 10^1^^4^ ions/cm^2^ in 11-µm-thick resist. Using 2-µm-thick resist and 2 MeV protons, structures of 110 nm were fabricated at CIBA-NUS [165]. Along with these resists Forturan, PDMS, and ma-N resists are also commercially available mentioned in Table 5.

#### 6.1.4. Resist for IPL

The commonly used resist for ion lithography is PMMA. Using PMMA resist of thickness 60 nm exposed to 50 keV Ga^+^ ions, 15 nm wide lines can be observed [168]. But PMMA is losing its importance due to its low etch resistance. Other resists are gaining importance with good contrast. AZ-5206 is a positive tone resist, exposed to H^+^ ions at a dose of 1.9 µC/cm^2^, gives a contrast of 3.3. SAL-601, a negative tone resist supplied by Shipley, exposed to H^+^ ions at a dose of 0.11 µC/cm^2^ gives a contrast value of 3.2. Contrast value 5.0 is obtained when HiPR-6512 is exposed to H^+^ ions with a dose of 1.0 µC/cm^2^ and exposed to He^+^ ions at the same dose. ARCH supplied by OCG exposed to H^+^ ions at a dose of 6.9 µC/cm^2^ gives a contrast value of 30.0 [169].

### 6.2. Mask

In ion beam lithography, ions such as H^+^, H^2+^, H^3+^, or He^+^ are used to expose the resist. Using light ions in the process has advantages of having high intrinsic resolution (10 nm), high resist sensitivity, very less or no proximity effect, enhances mask lifetime and reducing the device damage by using buffer layers. We can fabricate stencil mask of a large area with low distortion for demagnifying ion projection lithography technique. Progress of development in stencil masks for IPL was reported at MNE99 conference [170]. The development was based on a 150 mm SOI wafer. This solved the problem of distortion and complementary mask splitting. For the 200 mm stencil mask, membranes with 75 mm diameter have a design field of 50 × 50 mm^2^. By using a 4× optical reduction system, it can expose a field of 12.5 × 12.5 mm^2^. Die of dimension 25 × 25 mm^2^ can be printed with these four fields. According to the SIA roadmap, this field is equal to the size of a chip. One lithography layer of 256 Mb DRAM chip can be printed with this mask by repeating 32 times with a field of 110 × 110 mm^2^. Two 4 GB DRAM chips can be manufactured with this memory. A feature size of 50 nm can be obtained on the wafer by using this technology.

In several past years, development in the fabrication of stencil masks for proximity printing has taken place. Quantum effect and various microwave devices have been fabricated with the help of proximity printing at Sub-100 nm resolution with a field of 2 × 2 mm. High-resolution masks with thicknesses less than 0.5 µm are required.

### 6.3. Limitations

FIB has the same limitations as that of e-beam lithography. P-beam writing shows high throughput than the FIB. IPL improves the resolution and throughput significantly but the process of mask fabrication for IBL is very complex such as X-ray lithography. A high vacuum environment is required for the ion beam operation. Wafers are forced to bring in and out of the device during the process which consumes time. Due to charged particles, there remain chances of pattern blurring and pattern placement accuracy. Magnetic stages such as in optical lithography cannot be used for IBL. Ion beams can perturb the wafer structure and can create doping or other effects. Stencil mask technology used for complex patterning requires a complimentary mask. It increases the cost per level for mask fabrication. Moreover, there are very fewer commercial instruments available now for IBL. This technique requires much more development to be used for practical purposes.

## 7. Conclusions and Future Aspects

In the present work, advancement in the exposure tools, sources, resist, mask, and alignment marks for optical lithography, extreme ultraviolet lithography, electron beam lithography, and ion beam lithography has been discussed along with the process. Various new exposure tools have come into play with different field sizes, improving the resolution of the devices. Optical lithography has been used for feature size greater than 100 nm, below 100 nm difficulty of low resolution and throughput was obtained. Below 100 nm, next-generation lithography techniques have been represented which enhanced the resolution with their individually unique features. New resist materials with different doses showed feature sizes at the nanometer scale. EUVL achieved resolution up to 28 nm at a wavelength of around 13 nm. Then with the use of electron beam lithography feature size below 10 nm was achieved. E-beam is a maskless lithography technique reducing the steps in the patterning of the substrate. X-ray achieved a resolution up to 13 µm with a new set of tools. Then ion beam lithography has been discussed which achieved the feature size below sub-10 nm. With the help of double patterning and coherent lithography, feature size up to sub-1 nm can be obtained with IBL.

Optical lithography has limitations in the depth of focus, field size, and overlay accuracy. Resolution as low as 0.18 µm is not inconceivable with effective actinic wavelength reduction and the development of low k1 technologies. Better lenses, lower k1 technology, scanning or stitching can all be used to get around the field size restriction. Under ideal circumstances, overlay accuracy can be as low as 0.07 µm. Fundamental issues need to be resolved with EUV projection. Source, mask, and resist issue plague EUV projection. Technology development of high-NA EUV lithography is well underway, with insertion into HVM targeted for 2025. In many ways, high-NA EUV lithography is an evolutionary extension of 0.33 NA EUV lithography, but it will nevertheless be extremely challenging. For the optics, very large mirrors will need to be fabricated with an exceeding good surface figure and low roughness. Significant advances in mechatronics are needed to simultaneously meet throughput and overlay requirements. The creation of high-NA EUV exposure systems will be a remarkable engineering achievement. Mask and small-scale device fabrication are carried out with electron beam lithography. The ability to increase throughput in electron beam lithography is a key hurdle. Using a single fine beam to concurrently achieve higher resolution and throughput proved extremely difficult. The variable-shaped beam system and projection systems are created to solve this problem. The only method that appears to have shown every part of an entire system is X-ray lithography. The implementation of X-ray lithography, however, has been put off to the point where it may be viewed as a single-generation technology solution due to the industry’s resistance to accepting 1× mask-making and the high cost of entry with synchrotron radiation sources. Ion beam lithography owing to the new technique has the least developed tools. The basic issue with stencil masking in ion beam projection may necessitate the use of additional masks per set. The next ten years will be challenging as high-resolution lithography emerges as a prominent domain of nanotechnology.

## Figures and Tables

**Table 1:** Advancements in exposure tool of EUVL.

Tool Name	Developer	Year	EUV Source	NA	Reduction	Mirrors	Field Size (mm^2^)	Resolution (nm)	Ref.
ETS	EUV LLC	2001	LPP	0.1	4×	4	24 × 32.5	100	[42]
MS-13	EXITECH	2004	Xenon DPP	0.3	5×		0.6 × 0.2	32	[44]
HINA-3	NIKON	2005		0.3			0.3 × 0.5	30	[45]
AD Steppers	ASML	2006	Tin DPP	0.25	4×	6	26 × 33	50	[24]
EUV1	NIKON	2007	Xenon DPP	0.3	5×	6		25	[49]
NXE3100	ASML	2010		0.25				28	[47]
NXE3300B	ASML	2013		0.33				13 nm for single exposure and 9 nm for double exposure	[47]

**Table 2:** Parameters required for resist at the industrial level [55,56].

Parameters	Resolution	LWR	Sensitivity
**Dimensions**	<10 nm	<15%	<20 mJ/cm^2^

**Table 3:** Advanced resists for EBL technique.

Resist	Post Bake Exposure Temp. (°C)	Time of Developing (s)	Developing Solution	Contrast	Sensitivity (µC/cm^2^)	Resolution (nm)	Ref.
GMA-co-MMA-co-TPSMA			dimethylformamide		300	15	[100]
GMA-co-MMA-co-TPSMA	80	120	dimethylformamide		70	20	[100]
_40_XT		5	PEDOT:PSS (No dilution)	8 ± 2	8	95	[109]
_40_XT		120	PEDOT:PSS (40% dilution)	10 ± 0.3	7.5	80	[109]
MAPDST-MMA	100	120	TMAH	1.8	2.06	20	[103]
(P(HEMA-co-MAAEMA))			methanol	1.2	0.89	125	[110]
C_60_−(P(CMSx−HS))_2_			acetone		40	50	[111]
(P(HEMA-co-MAAEMA))			amyl acetate	4.77	172	10	[110]
HafSOx			TMAH	2.5	21	7	[106]
ZircSOx			TMAH	2.6	7.6	7	[106]

**Table 4:** Different resists for X-ray lithography technique.

Resist Name	Nature	Required Incident Dose (J cm^−2^)	Resolution	Ref.
PMMA	Positive	0.33	350 Å	[132]
PMMA	Positive	0.5	50 Å	[133]
TIP(MMA-MMA)	Positive	0.024	1000 Å	[132]
CoP (MMA-MAA)	Positive	0.05	500 Å	[134]
DCIPA	Negative	0.0078	0.5 µm	[132]
Epoxidized polybutadiene	Negative	0.0015	1 µm	[135]

**Table 5:** Resists for proton beam technique.

Resist	Type of Resist	Fluence (ions/cm^2^)	Energy of Proton	Aspect Ratio	Smallest Feature Written	Ref.
PMMA	Positive	5.0–9.4 × 10^13^	2	100	20–30 nm	[156]
SU-8	Negative	1.9 × 10^13^	1	166	60 nm	[146]
HSQ	Negative	1.9–13 × 10^13^	2	40	19 nm	[163]
TADEP	Negative	1.6–15 × 10^13^	2	18	110 nm	[165]
AGAR	Positive	3.0 × 10^14^	2.25	0.3	15 µm	[160]
Forturan	Positive	6.3 × 10^11^	2	13.3	3 µm	[166]
PDMS	Negative	0.13–40 × 10^13^	1	1.3	10 µm	[167]
ma-N 2401	Negative	2.5 × 10^13^	1	1.6	60 nm	[166]

## Data Availability

Not applicable.
